# Elucidating the cellular mechanism for E2-induced dermal fibrosis

**DOI:** 10.1186/s13075-021-02441-x

**Published:** 2021-02-27

**Authors:** DeAnna Baker Frost, Alisa Savchenko, Adeyemi Ogunleye, Milton Armstrong, Carol Feghali-Bostwick

**Affiliations:** 1 Department of Medicine, Division of Rheumatology, Medical University of South Carolina, Charleston, USA; 2 Division of Plastic Surgery, University of North Carolina, Chapel Hill, USA; 3 Department of Surgery, Division of Plastic Surgery, Medical University of South Carolina, Charleston, USA

**Keywords:** Estradiol, Fibrosis, Dermal fibroblasts, Skin, TGFβ

## Abstract

**Background:**

Both TGFβ and estradiol (E2), a form of estrogen, are pro-fibrotic in the skin. In the connective tissue disease, systemic sclerosis (SSc), both TGFβ and E2 are likely pathogenic. Yet the regulation of TGFβ in E2-induced dermal fibrosis remains ill-defined. Elucidating those regulatory mechanisms will improve the understanding of fibrotic disease pathogenesis and set the stage for developing potential therapeutics. Using E2-stimulated primary human dermal fibroblasts in vitro and human skin tissue ex vivo, we identified the important regulatory proteins for TGFβ and investigated the extracellular matrix (ECM) components that are directly stimulated by E2-induced TGFβ signaling.

**Methods:**

We used primary human dermal fibroblasts in vitro and human skin tissue ex vivo stimulated with E2 or vehicle (ethanol) to measure *TGFβ1* and *TGFβ2* levels using quantitative PCR (qPCR). To identify the necessary cell signaling proteins in E2-induced *TGFβ1* and *TGFβ2* transcription, human dermal fibroblasts were pre-treated with an inhibitor of the extracellular signal-regulated kinase/mitogen-activated protein kinase (ERK/MAPK) pathway, U0126. Finally, human skin tissue ex vivo was pre-treated with SB-431542, a TGFβ receptor inhibitor, and ICI 182,780, an estrogen receptor α (ERα) inhibitor, to establish the effects of TGFβ and ERα signaling on E2-induced collagen 22A1 (*Col22A1*) transcription.

**Results:**

We found that expression of *TGFβ1*, *TGFβ*2, and *Col22A1*, a TGFβ-responsive gene, is induced in response to E2 stimulation. Mechanistically, *Col22A1* induction was blocked by SB-431542 and ICI 182,780 despite E2 stimulation. Additionally, inhibiting E2-induced ERK/MAPK activation and early growth response 1 (*EGR1*) transcription prevents the E2-induced increase in *TGFβ1* and *TGFβ2* transcription and translation.

**Conclusions:**

We conclude that E2-induced dermal fibrosis occurs in part through induction of *TGFβ1,* 2, and *Col22A1*, which is regulated through EGR1 and the MAPK pathway. Thus, blocking estrogen signaling and/or production may be a novel therapeutic option in pro-fibrotic diseases.

## Background

Transforming growth factor beta (TGFβ) is central to the production of extracellular matrix (ECM) [1], which promotes wound healing [2] but can also lead to organ fibrosis, especially in the connective tissue disease, systemic sclerosis (SSc) [3–6]. Several studies focus on TGFβ as an inducer of fibrosis [1], but few studies explore the regulation of TGFβ. Because TGFβ is a major inducer of fibrosis, its inhibition was the emphasis for a potential pharmacologic intervention in SSc, without success [7]. Since there are limited treatment options for SSc, it is important to understand the regulation of TGFβ isoforms to understand SSc disease pathogenesis and develop potential therapeutics.

Like TGFβ, the steroid hormone estrogen augments fibrosis. Because SSc has a female predominance [8], estrogen is hypothesized to be pathogenic in fibrosis and to contribute to sex differences noted in animal models of fibrosis. Female rats treated with bleomycin had more severe fibrosis and higher mortality than male rats [9]. In radiation-induced lung fibrosis, rats had less lung fibrosis if treated with an aromatase inhibitor that blocks estrogen production [10]. Likewise, mice with a heritable form of pulmonary hypertension that were treated with either an estrogen receptor inhibitor or an aromatase inhibitor showed significant disease improvement [11]. These results imply estrogen is influential in lung fibrosis and its blockade is a conceivable therapeutic target.

Estradiol (E2) is the most bioactive and abundant form of estrogen in non-pregnant women [12]. In the skin, E2 promotes ECM production. Women on hormone replacement therapy (HRT) have increased dermal thickness and collagen content than women not taking HRT [13–18]. Our group has shown that E2 levels are increased in post-menopausal women with SSc, and E2 induced dermal thickening and increased fibronectin (FN) in an ex vivo human skin organ culture model and in primary human dermal fibroblasts [19]. Elevated E2 levels in patients with SSc are associated with reduced survival [20], suggesting a role for E2 in SSc-fibrosis.

As with TGFβ, most studies regarding E2-induced fibrosis have centered on ECM production. However, few experiments have focused on the production of E2-induced pro-fibrotic mediators or the transcriptional regulation of these proteins. E2 induced the pro-fibrotic mediators connective tissue growth factor (CTGF) in rats [21] and TGFβ1 in a model of wound healing [22, 23]. Yet, the cell signaling molecules used by E2 to induce TGFβ1 remain undefined. In this study, we seek to characterize E2-induced dermal fibrosis by describing the underlying cellular mechanisms that promote fibrosis.

## Methods

### Ex vivo human skin organ culture

We received skin samples from healthy donors of various ages who underwent skin-resection procedures in the Division of Plastic Surgery at the Medical University of South Carolina under an approved IRB protocol. The demographic information of the donors is summarized in Table 1. The ex vivo human skin organ culture model was used as previously described [19, 24, 25]. For experimentation, we used a six-well tissue culture dish (Costar, Corning, NY) with six 3-mm punches/well placed dermal side down in serum-free, phenol red-free DMEM (Corning, Corning, NY). In all experiments, skin punches and supernatants were harvested at the specified times and stored at − 80 °C until further evaluation.

**Table 1:** Demographics of the human skin donors used in the human skin organ culture model ex vivo experiments

ID	Age	Sex	Anatomic location
10	25	F	Breast
30	33	F	Abdomen
33	35	F	Abdomen
216	34	F	Abdomen
217	46	F	Arm
218	52	F	Abdomen
220	36	F	Buttocks
225	43	F	Abdomen
227	51	F	Abdomen
230	37	Unknown	Abdomen
231	Unknown	Unknown	Abdomen
237	38	Unknown	Abdomen
268	36	M	Abdomen
275	35	M	Abdomen
277	66	M	Abdomen
278	36	F	Back
279	39	F	Abdomen
287	51	F	Abdomen
323	Unknown	Unknown	Unknown

### Cell culture

Primary fibroblasts were isolated from human dermal tissue using the previously described outgrowth method [19]. Primary dermal fibroblasts (passages 3–8) were plated in six-well culture dishes (Costar, Corning, NY) at a concentration of 2.0 × 10^5^ cells/well. Prior to stimulation, the cells were serum-starved in serum-free, phenol red-free DMEM (Hyclone, South Logan, UT). Cells were then treated with 10 μM of a mitogen-activated protein kinase/extracellular signal-regulated kinase (MAPK/ERK) inhibitor, U0126 [26], 100 nM of the estrogen receptor alpha (ERα) inhibitor fulvestrant (ICI 182,780) or 10 μM of the TGFβ receptor inhibitor, SB-431542 [27], 1 h before stimulation with E2 or ethanol (ETOH) as vehicle. U0126, SB-431542, and E2 were obtained from MilliporeSigma (St. Louis, MO), ICI 182,780 from Tocris (Minneapolis, MN), and the ETOH from Hyclone (South Logan, UT). For subcellular fractions, cells were grown in 10-cm culture dishes and maintained in complete media (DMEM supplemented with 1x antibiotic and antimycotic) from Hyclone. Once 90–95% confluent, the cells were serum-starved for at least 8 h in phenol red-free media then stimulated with ETOH or E2 for the indicated timepoints. The subcellular fractions were isolated using a subcellular protein fractionation kit for cultured cells (Thermofisher Scientific, Rockford, IL).

### Measurement of steady-state mRNA levels

Total RNA was isolated from human skin punches through homogenization using TRIzol (Invitrogen, Carlsbad, CA), with further purification using a RNeasy isolation kit (Qiagen, Hilden, Germany). RNA from primary dermal fibroblasts was isolated with a RNeasy isolation kit. Steady-state mRNA levels were measured using quantitative PCR (qPCR) and levels are shown as fold change over vehicle following normalization of signal to *B2M* and *GAPDH*. Primers specific for *FN*, *Col22A1*, *TGFβ1, TGFβ2, TGFβ3, collagen IA2 (Col IA2), collagen IIIA1 (Col IIIA1)*, and *early growth response 1 (EGR1)* were all purchased from Thermofisher Scientific (Rockford, IL).

### siRNA transfection

Primary dermal fibroblasts were plated at 1.5 × 10^5^ cells/mL in six-well culture dishes. After reaching 70% confluency, the cells were transfected with siRNA targeted to *EGR1* or a negative control pool (composed of four siRNAs) of non-targeting siRNA (CTL) (Horizon Discovery, Cambridge, UK) at 100 nM for 24 h and then serum-starved in phenol red-free media for 8 h before treatment with ETOH or E2 for 24 h.

### Immunoblot analysis

Whole-cell protein lysates or cellular fractions from dermal fibroblasts were subjected to immunoblot analysis. Equal volumes (20 μL/lane) were resolved on a 10% SDS PAGE gel and transferred to a nitrocellulose membrane (GE Healthcare Life Sciences, Pittsburgh, PA). Following blocking with 5% non-fat dry milk, membranes were incubated with antibodies against phosphorylated ERK (Rabbit polyclonal, catalog # 9101, 1:1000 dilution, Cell Signaling Technology, Danvers, MA), total ERK (Rabbit monoclonal, catalog # 4695 S, 1:1000 dilution, Cell Signaling Technology, Danvers, MA), TGFβ1, (Rabbit monoclonal, clone EPR18163, 1:1000 dilution, Abcam, Cambridge, UK), EGR1 (Rabbit monoclonal, clone 15F7, 1:1000 dilution, Cell Signaling Technology, Danvers, MA), or GAPDH (Mouse monoclonal, catalog # sc-47,724, 1:5000 dilution, Santa Cruz, Santa Cruz, CA), followed by horseradish peroxidase-conjugated secondary antibodies. After being washed, immunoblots were developed with chemiluminescence reagents according to the manufacturer’s protocol (Pierce, Rockford, IL). Total MAP kinases were examined in the same immunoblots as their phosphorylated forms. Signal intensities of the phosphorylated MAP kinase bands were quantitated by densitometry, and the results, which were normalized against the intensities of the corresponding total MAP kinase bands in each sample, were expressed as the magnitude of increase compared with controls. Densitometry was calculated using Image J.

### Statistical analysis

All data were tested for normal distribution using the Shapiro-Wilk normality test. If the data were normally distributed, a one-way ANOVA with Sidak’s multiple comparisons post hoc test or paired, two-sample *t* test were used to determine statistical significance. For non-normally distributed data, non-parametric one-way ANOVA using Dunn’s multiple comparisons post hoc test or Wilcoxon matched-pairs signed rank tests were used to determine statistical significance, which was defined as a *p* value ≤ 0.05 using GraphPad Prism v.9.

## Results

### Demographics

Table 1 includes the demographic information of healthy subjects who donated skin tissue used in the ex vivo human skin organ culture model experiments. The majority of the donors (63%) were women, with the donated tissue primarily isolated from the abdomen (74%).

### E2 increased transcription of pro-fibrotic mediators in vitro

**Fig. 1:**
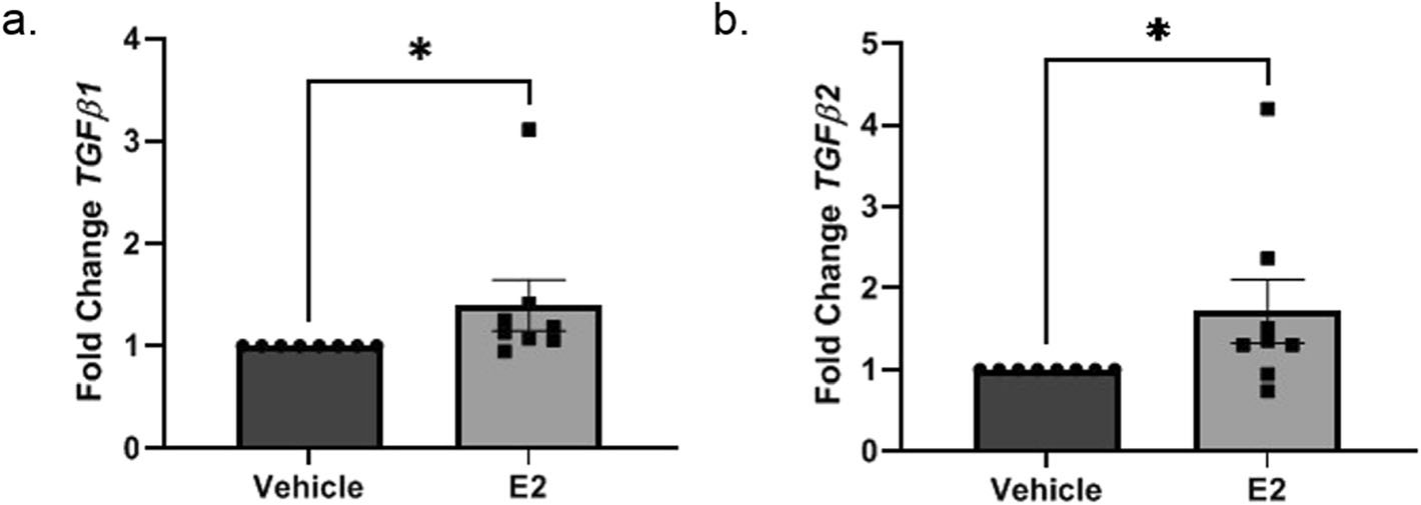
E2-induced *TGFβ1* and *TGFβ2* transcription in vitro. Steady-state mRNA levels 24 h post vehicle vs. E2 stimulation in vitro. Steady-state mRNA levels of *TGFβ1* (**a**) and *TGFβ2* (**b**). Normalized to *B2M*. Data shown are from 8 independent experiments using dermal fibroblasts from 8 different donors. Bars = mean ± SEM. Statistical test: Two-tailed, Wilcoxon matched-pairs signed rank test.*p ≤ 0.05

Pro-fibrotic mediators and ECM components were measured in primary human dermal fibroblasts post E2 stimulation. *TGFβ1* and *TGFβ2* transcript levels were 1.4- and 1.7-fold higher in E2-stimulated cells, respectively, than vehicle-treated cells at 24 h, which reached statistical significance (Fig. 1a, b). *TGFβ1* and *TGFβ2* transcript levels showed an increasing trend 48 h post E2 stimulation (Supplementary Figure 1, Additional file 1). However, *TGFβ3* did not increase significantly at the same time point (Additional file 2).

### E2 activated ERK1/2 in primary human dermal fibroblasts

**Fig. 2:**
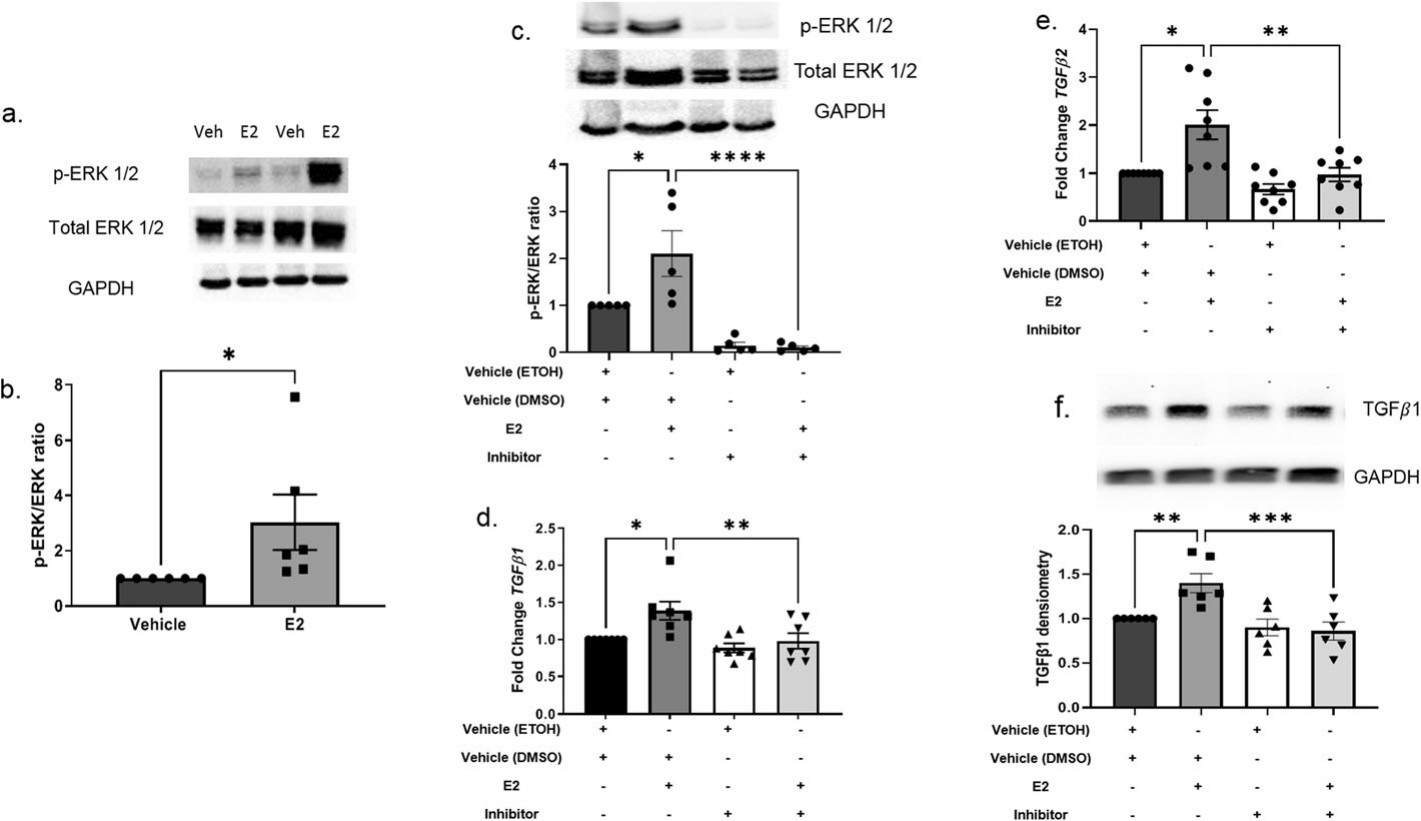
E2-induced activation of pERK and its effects on *TGFβ1* and *TGFβ*2 transcription and translation in vitro. **a** Representative immunoblot of extracellular signal-regulated kinase 1/2 (ERK1/2) activation 1 h post E2 stimulation in vitro. **b** Densitometry of p-ERK1/2 to total ERK1/2 ratio. **c** Representative immunoblot and densitometry of p-ERK1/2 to total ERK ratio after 1 h pre-treatment with the mitogen-activated protein kinase (MAPK)/ERK inhibitor, U0126, and subsequent 1 h of E2 stimulation. **d**, **e** Steady-state transcript levels of *TGFβ1* (**d**) and *TGFβ2* (**e**) in primary human dermal fibroblasts following a 1 h pre-treatment with U0126 and subsequent 24 h post E2 stimulation. Representative immunoblot of TGFβ1 (**f**) in primary human dermal fibroblast lysates during a 1 h pre-treatment with U0126 and subsequent 48 h post E2 stimulation. Ethanol, ETOH; estradiol, E2; glyceraldehyde 3-phosphate dehydrogenase, GAPDH; dimethyl sulfoxide, DMSO. Normalized to GAPDH (**b**, **c**, **f**) or *B2M* (**d**, **e**). Bars = mean ± SEM. Data shown is from ≥ 6 independent experiments using dermal fibroblasts from ≥ 6 different donors. Statistical analysis: Two-tailed, Wilcoxon matched-pairs signed rank test (**b**) and one-way ANOVA with Sidak’s multiple comparisons post hoc test (**c**–**e**). *p ≤ 0.05, **p ≤ 0.01, ***p ≤ 0.005, ****p ≤ 0.0001

E2 induces MAPK cell signaling in uterine leiomyomas [28]. Therefore, we investigated if E2-treated primary human dermal fibroblasts induce ERK1/2 phosphorylation, signifying MAPK activation. After 1 h, E2 stimulation led to a 3.7-fold greater induction in phosphorylated/activated ERK1/2 compared to total ERK1/2 (*p* < 0.05) (Fig. 2a, b). We did not observe significant increases in phosphorylated ERK1/2 at earlier time points (Additional file 3). Other MAPK family members, including p-38 and JNK, were examined and were not activated (Additional file 4).

### MAPK/ERK inhibition reduced E2-induced *TGFβ1* and *TGFβ2* transcription and translation

We showed that E2 induced *TGFβ1* and *TGFβ2* transcription and ERK1/2 activation in vitro. Next, we tested the effects of blocking upstream ERK activation on *TGFβ1* and *TGFβ2* transcription in primary human dermal fibroblasts, using the inhibitor U0126 [26]. We first confirmed that U0126 inhibited E2-induced ERK activation (Fig. 2c). Pre-treatment with the inhibitor 1 h before E2 stimulation prevented induction of *TGFβ1* and *TGFβ2* transcription after 24 h and TGFβ1 translation after 48 h, compared to E2 and vehicle (Fig. 2d–f).

### EGR1 regulated E2-induced *TGFβ1* and *TGFβ2* transcription

**Fig. 3:**
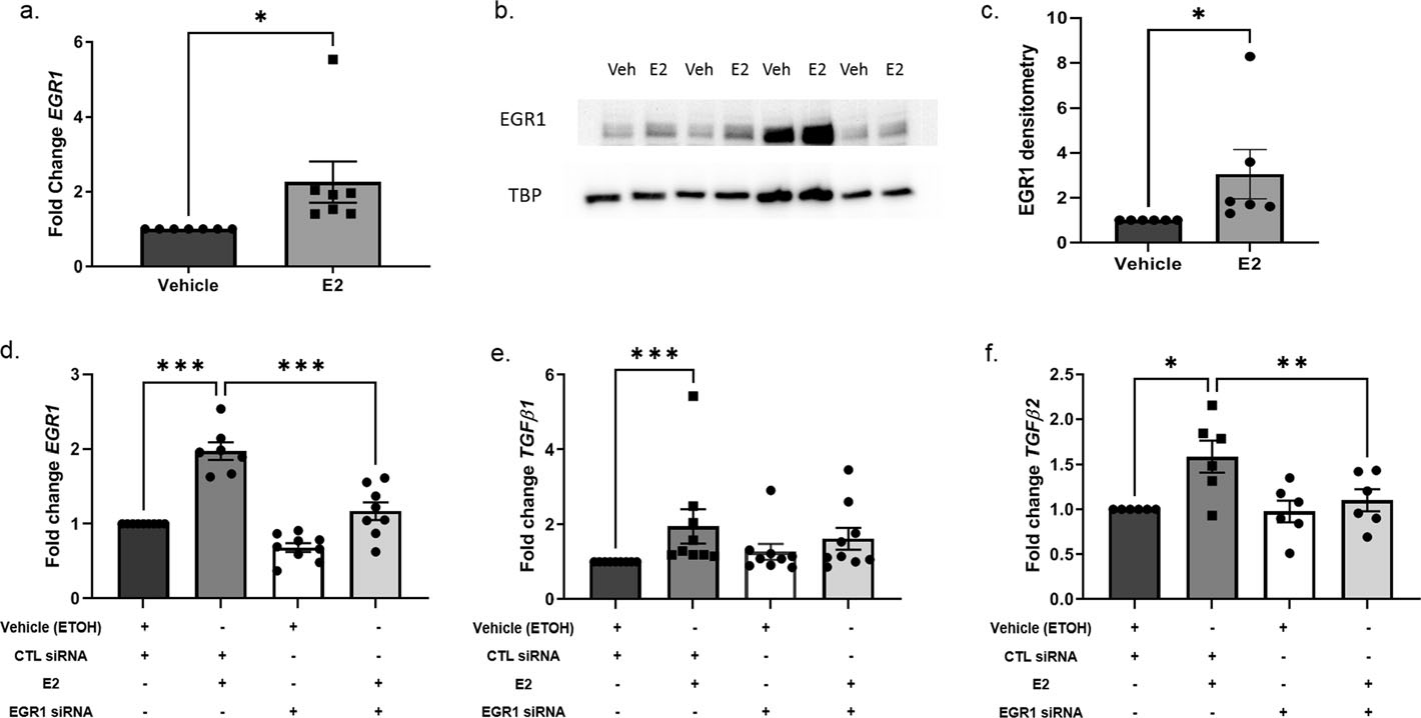
E2-induced EGR1 transcription and translation and its effects on *TGFβ1* and *TGFβ*2 transcription in vitro. **a** Steady-state transcript levels of *EGR1* 6 h post vehicle vs. E2 stimulation in vitro. **b** Representative immunoblot of EGR1 protein 16 h post  E2 stimulation. **c** Densitometry of EGR1 immunoblot. **d**–**f** Steady-state transcript levels of *EGR1* (**d**), *TGFβ1* (**e**), and *TGFβ2* (**f**) in primary human dermal fibroblasts transfected with siRNA against *EGR1* 24 h post E2 stimulation. Control, CTL. Normalized to *B2M* (**a**, **d**–**f**) or Tata-binding protein (TBP) (**c**). Bars = mean ± SEM. Data from **a**–**f** includes ≥ 6 independent experiments using dermal fibroblasts from ≥ 6 different donors. Statistical tests: Wilcoxon matched-pairs signed rank test (**a**, **c**) and parametric, one-way ANOVA with Sidak’s multiple comparisons post hoc test (**d**, **f**), and non-parametric one-way ANOVA using Dunn’s multiple comparisons (**e**). *p ≤ 0.05, **p ≤ 0.01, ***p ≤ 0.005

Several transcription factor binding sites are located in the promoter region of the TGFβ isoforms [29, 30], including EGR1 [31]. EGR1 is a known transcription factor that mediates fibrosis [32–34]. Because E2 is known to activate transcription factors [35], we investigated whether E2 induces EGR1 in primary human dermal fibroblasts. While *EGR1* transcript levels were not significantly increased 30 min post E2 stimulation (Supplementary Figure 2, Additional file 5), its transcription and translation were significantly increased 6 and 16 h after E2 stimulation, respectively (Fig. 3a–c). *EGR1* transcript levels remained elevated 8 h post E2 stimulation (Supplementary Figure 2, Additional file 5). We then silenced *EGR1* to investigate whether EGR1 regulates E2-induced *TGFβ1* and *TGFβ2* transcription in primary human dermal fibroblasts. Transfection of dermal fibroblasts with *EGR1*-targeted siRNA significantly diminished levels of *EGR1* and *TGFβ2* in E2-stimulated cells, with a trend for *TGFβ1* reduction (Fig. 3d–f).

### E2 increased transcription of ECM components and pro-fibrotic mediators ex vivo

**Fig. 4:**
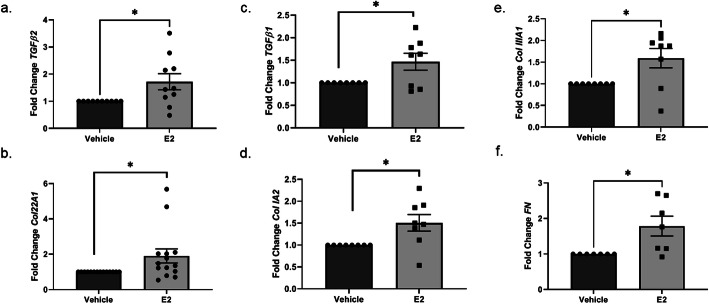
E2-induced transcription of ECM components and pro-fibrotic mediators ex vivo. Steady-state mRNA levels of *TGFβ2* (**a**), *Col22A1* (**b**), *TGFβ1* (**c**), *Col I IA2* (**d**), *Col IIIA1* (**e**), and *FN* (**f**) at 24 (**a**), 48 (**b**, **c**), or 72 (**d**–**f**) h post vehicle vs. E2 stimulation of human skin ex vivo. Bars = mean ± SEM. Normalized to *B2M* (**a**–**e**) and *GAPDH* (**f**). Data shown are from ≥ 8 independent experiments using human skin tissue from ≥ 8 different donors. Statistical test: Two-tailed, parametric, paired *t* test (**a**–**d**, **f**), Wilcoxon matched-pairs signed rank test (**e**). *p ≤ 0.05

Even though both in vitro and in vivo evidence suggests that E2 induces FN protein levels ex vivo [19], its effects on the steady-state mRNA levels of other ECM products are unknown. We measured steady-state mRNA levels of known genes implicated in fibrosis in an ex vivo human skin organ culture model 24, 48, and 72 h after E2 stimulation. At 24 h post E2 stimulation, *TGFβ2* transcript levels increased in E2-stimulated vs. vehicle-treated skin (Fig. 4a), and the effect subsided 48 h post E2 stimulation (Additional file 6). Col22A1 is an early, TGFβ*-*responsive gene that contributes to the ECM [36]. E2 also induced *Col22A1* transcription at 48 h post stimulation (Fig. 4b), not at 72 h (Additional file 6). Interestingly, similar increases were observed for steady-state mRNA levels of *TGFβ1*, albeit at 48 h post E2 stimulation (Fig. 4c), while *Col IA2, Col IIIA1*, and *FN* transcription was significantly increased at 72 h (Fig. 4d–f) post stimulation, but not 24 or 48 h (Additional file 6). There was not a statistically significant induction in the steady-state transcript levels of *Col IA1, connective tissue growth factor (CTGF),* or  *alpha smooth muscle actin (alpha-SMA)* (Additional file 7).

### Inhibiting TGFβ1 signaling decreased E2-induced *Col22A1* transcription ex vivo

**Fig. 5:**
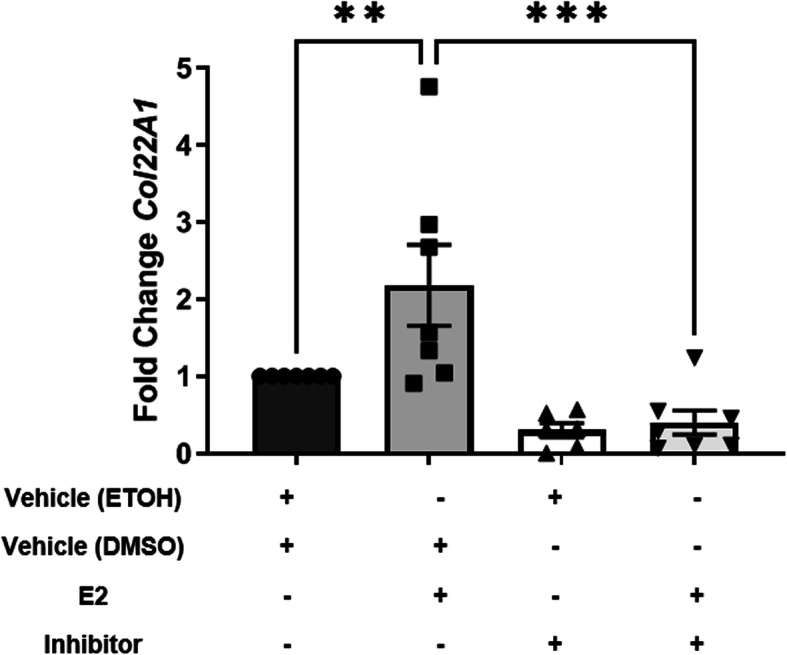
*Col22A1* transcription post E2 stimulation and TGFβ1 signaling blockade ex vivo. Steady-state transcript levels of *Col22A1* during a 1 h pre-treatment with the TGFβ type I receptor inhibitor, SB-431542, 48 h post E2 stimulation, ex vivo. Ethanol, ETOH; estradiol, E2, dimethyl sulfoxide, DMSO, normalized to *B2M*. Bars = mean ± SEM. Data shown are from ≥ 6 independent experiments using human skin from ≥ 6 different donors. Statistical test: Parametric one-way ANOVA with Sidak’s multiple comparisons post hoc test. **p ≤ 0.01, ***p ≤ 0.005

E2 induced *TGFβ1* and *TGFβ2* transcription earlier than other ECM components, suggesting that the ECM is induced through TGFβ1 and TGFβ2. To investigate whether TGFβ signaling mediates the increase in *Col IA2, Col IIIA1, FN,* and *Col22A1* post E2 stimulation, we pre-treated human ex vivo skin samples with SB-431542, a type 1 TGFβ receptor inhibitor [27], before stimulating with E2 for an additional 24, 48, or 72 h. SB-431542 reduced steady-state mRNA transcript levels of *Col22A1* despite E2 treatment (Fig. 5), but not *Col IA2, Col IIIA1*, and *FN* transcription to below those of the E2-treated skin samples suggesting, that E2 induction of *Col22A1* is via TGFβ.

### Inhibiting E2 signaling through estrogen receptor alpha inhibited *Col22A1* transcription ex vivo

**Fig. 6:**
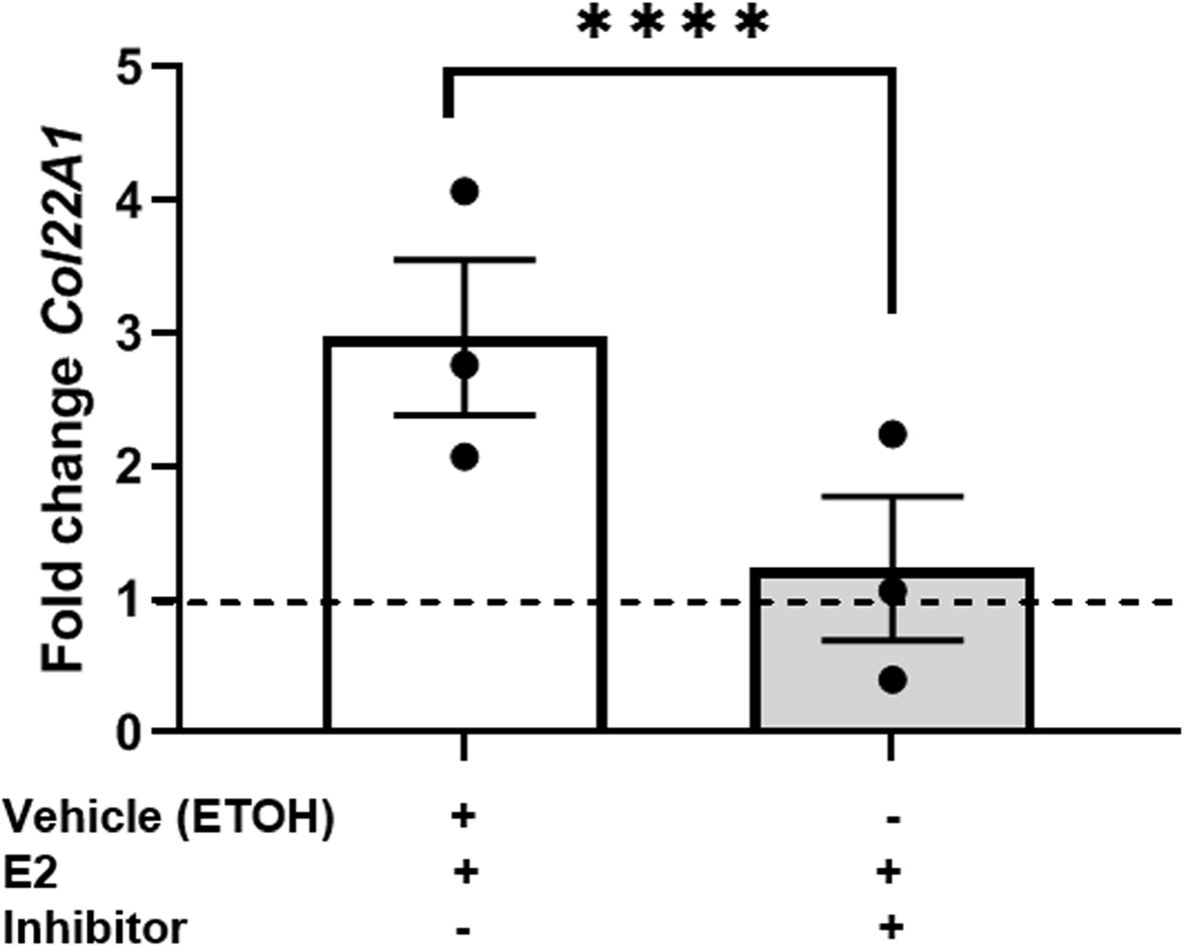
*Col22A1* transcription post E2 signaling blockade ex vivo. Steady-state transcript levels of *Col22A1* following a 1 h pre-treatment with the ERα inhibitor, fulvestrant, 24 h post E2 stimulation of human skin ex vivo. Dotted line indicates normalization to baseline treatment with ETOH alone. Ethanol, ETOH; estradiol, E2. Normalized to *B2M*. Bars = mean ± SEM. Data shown are from human skin tissue of one donor performed in 3 independent experiments. Statistical test: Two-tailed, parametric, paired *t* test. ****p ≤ 0.001

We demonstrated that E2-induced FN production and dermal thickness ex vivo are inhibited by fulvestrant (ICI 182,780), which inhibits estrogen signaling through one of its major receptors, estrogen receptor alpha (ERα) [19]. We tested if E2 inhibition through ERα blockade would prevent the E2-induced increase in *Col22A1* transcription ex vivo. After 1 h pre-treatment of human tissue with the ERα inhibitor, ICI 182,780, then subsequent ETOH and E2 treatment, E2-induced *Col22A1* transcription was significantly decreased (Fig. 6).

## Discussion

Both E2 and TGFβ1 are central to ECM production in the skin; however, little is known about the cellular mechanisms underlying E2-induced dermal fibrosis or the transcriptional and translational regulation of TGFβ. We are the first to report that, in the skin, E2 induces TGFβ1 and TGFβ2 through ERα signaling, MAPK activation, and EGR1 induction. TGFβ1 and TGFβ2, in turn, induce Col22A1. Based on these observations, we propose a cellular mechanism that mediates E2-induced dermal fibrosis.

Col22A1 has been identified in patients with SSc and is known to contribute to fibrosis. Whole exome sequencing of patients with diffuse cutaneous SSc showed an enrichment of genes in the ECM pathway, including Col22A1 [37]. Additionally, whole exome sequencing of African American patients with SSc identified* Col22A1* as a rare variant that may increase African American susceptibility to SSc [38]. To characterize the regulation of Col22A1, our group reported that primary human dermal fibroblasts from patients with SSc and healthy controls release Col22A1 in direct response to TGFβ1 stimulation. We also showed that Col22A1 mediates the transition of fibroblasts to myofibroblasts [36]. These data indicate that Col22A1 is detected in patients with SSc and likely contributes to fibrosis. Our current study extends the understanding of Col22A1 induction by demonstrating that it can occur in direct response to a pro-fibrotic mediator that induces TGFβ, namely E2. Thus, E2 contributes to dermal fibrosis through inducing Col22A1.

We show that both *TGFβ1* and *TGFβ2* are induced by E2, as they are in a wound healing model [22, 23], and both likely contribute to dermal fibrosis. Even though the *TGFβ1* and *TGFβ2* isoforms are encoded by different genes, both use the same signaling receptors and cascades [39–41] and likely contribute to E2-induced *Col22A1* transcription.

This report also extends the number of E2-induced ECM proteins and mediators known to impact dermal fibrosis to include *Col IA2*, *Col IIIA1*, *Col22A1*, *TGFβ1,* and *TGFβ2* in our ex vivo human skin organ culture model. Yet, *Col IA2* and *Col IIIA1* steady-state transcript levels were not increased in primary dermal fibroblast single-cell culture in vitro. The discrepancy between ex vivo and in vitro results may be explained by the inclusion of other cell types in ex vivo skin tissue, such as keratinocytes, which are responsive to E2 and can contribute to ECM formation [42–44].

While primary human dermal fibroblasts produce less *Col IA1*, *Col IA2, FN,* and *Col22A1* when pre-treated with the TGFβ receptor inhibitor SB-431542 before stimulation with TGFβ1 [36, 45], E2-induced *Col IA2, Col IIIA1,* and *FN* transcription was not prevented by pre-treatment with the inhibitor. This is likely because E2 can induce other pro-fibrotic mediators to influence ECM production. Our unpublished findings suggest that E2 increases the transcript and protein levels of the pro-fibrotic cytokine IL-6, which can then increase collagen and FN levels through trans-signaling [46]. IL-6 also activates STAT3, which promotes *Col IA2* synthesis post-transcriptionally [47]. Thus, E2-induced fibrosis likely occurs through both the non-canonical TGFβ-ERK and IL-6-STAT3 signaling pathways.

We investigated whether the MAPK pathway, which is central to E2-induced Col22A1 signaling, regulates TGFβ1 and TGFβ2. We report that E2-induced ERK1/2 phosphorylation in primary human dermal fibroblasts is vital to TGFβ1 and TGFβ2 expression, since blockade of the MAPK/ERK pathway was inhibitory. In apoptotic cells, TGFβ transcriptional and translational regulation also occurs through the MAPK pathway, in addition to the RhoA and PI-3K/AKT pathways [48]. Specifically, TGFβ1 synthesis is induced by activation of CD36 on macrophages in response to apoptotic cells [49]. Further studies are needed to elucidate the role of CD36 in E2-induced TGFβ1 and TGFβ2 transcription and translation in primary human dermal fibroblasts.

Transcription factors are crucial to comprehend E2-induced TGFβ1 and TGFβ2 transcription. EGR1 is a candidate transcription factor in this pathway because it has been reported downstream of TGFβ1 signaling in human dermal fibroblasts [32], and the EGR1-TGFβ1 relationship is important in a murine model of pulmonary fibrosis [50]. We report that EGR1 is also upstream of E2-induced *TGFβ1* transcription in primary human dermal fibroblasts, which is not surprising as EGR1 binds to and activates the TGFβ1 promoter [31] and exogenous expression of *EGR1* in the cancer cell line HT1080 led to increased secretion of TGFβ1 [51]. Additionally, EGR1 increased E2-induced *TGFβ2* transcription in primary dermal fibroblasts, which parallels the findings in experiments focused on tendon repair [52, 53].

ERα is one of the classical estrogen receptors involved in E2 signaling [54]. Fulvestrant is FDA-approved for use in breast cancer and is effective at ameliorating bleomycin-induced lung fibrosis [55]. We extend those findings in this report to suggest that ERα signaling blockade can also prevent E2-induced *Col22A1* transcription ex vivo*.* These results, in conjunction with our previous findings showing that circulating E2 levels are elevated in patients with SSc [20] suggest that fulvestrant is a promising therapeutic option in patients with SSc.

## Conclusions

Dermal fibrosis is a key feature of some pro-fibrotic diseases, such as SSc, making it critical that we understand its underlying mechanism. Here, we suggest a cell signaling mechanism for E2-induced dermal fibrosis and TGFβ regulation. We found that E2-induced TGFβ1 and TGFβ2 directly increased Col22A1 and contributed to ECM accumulation. Currently, several FDA-approved medications exist that inhibit E2 signaling and production. However, these medications have been limited to use in hormonal cancers. Our data suggest that therapies that inhibit E2 signaling may reduce dermal fibrosis and present an interesting treatment alternative in pro-fibrotic diseases.

## Supplementary Information

**Additional file 1: a-b.** Steady-state mRNA levels of *TGFβ1* and *TGFβ2* 48 h post vehicle vs. E2 stimulation in vitro. Steady state mRNA levels of *TGFβ1* (a), *TGFβ2* (b). Normalized to *B2M*. Data shown are from ≥5 independent experiments using dermal fibroblasts from 5 different donors. Bars = mean +/− SEM. Statistical test: Two-tailed, parametric, paired t-Test.

**Additional file 2. **Steady-state mRNA levels of *TGFβ3* 24 h post vehicle vs. E2 stimulation in vitro*.* Normalized to *B2M*. Data shown are from ≥5 independent experiments using dermal fibroblasts from 5 different donors. Bars = mean +/− SEM. Stastical test: Two-tailed, parametric, paired t-Test.

**Additional file 3.** Immunoblot of p-ERK1/2:total ERK after 15 or 30 min of E2 stimulation. Data shown are from 2 independent experiments using dermal fibroblasts from 2 different donors.

**Additional file 4.** Immunoblots of p-SAPK/JNK:total SAPK/JNK (a) and p-p38:total p-p38 (b) 15 or 30 min or 1 h post E2 stimulation in vitro. Data shown are from ≥2 independent experiments using dermal fibroblasts from 2 different donors.

**Additional file 5. **Steady-state transcript levels of *EGR1* 30 min (a) and 8 h (b) postvehicle vs. E2 stimulation in vitro. Normalized to *GAPDH*. Data shown are from 5 independent experiments using dermal fibroblasts from 5 different donors. 2 donors were measured in triplicate in (b). Bars = mean +/− SEM, Statistical test: Two-tailed, parametric, paired t-Test. *p ≤ 0.05

**Additional file 6. **Steady-state mRNA levels of *TGFβ2* (a), *Col22A1* (b), *TGFβ1* (c), *Col I IA2* (d), *Col IIIA1* (e), and *FN* (f) ex vivo. Measured at 48/72 (a), 72 (b), 24/72 (c), 24/48 (d-f) hours post vehicle vs. E2 stimulation ex vivo. Bars = mean +/− SEM. Normalized to *B2M* (a-e) and *GAPDH* (f). Data shown are from ≥8 independent experiments using dermal fibroblasts from ≥8 different donors. Statistical test: Two-tailed, parametric, paired t-Test (a-d, f) and Wilcoxon matched-pairs signed rank test (e).

**Additional file 7. **Steady-state mRNA levels of *alpha SMA* (a), *CTGF* (b), *Col IA1* (c) ex vivo. Measured at 24–72 h (a-b) and 24–48 h (c) hours post vehicle vs. E2 stimulation ex vivo. Bars = mean +/− SEM. Normalized to *B2M* (a-c). Data shown are from ≥6 independent experiments using dermal fibroblasts from ≥6 different donors. Statistical test: Two-tailed, parametric, paired t-Test.

## Data Availability

Not applicable.

## Abbreviations

E2: Estradiol
ETOH: Ethanol
DMSO: Dimethyl sulfoxide
SSc: Systemic sclerosis
TGFβ1: Transforming growth factor β1
TGFβ2: Transforming growth factor β2
qPCR: Quantitative PCR
Col IA2: Collagen IA2
Col IIIA1: Collagen IIIA1
Col22A1: Collagen 22A1
ECM: Extracellular matrix
ERK: Extracellular signal-regulated kinase
MAPK: Mitogen-activated protein kinase
EGR1: Early growth response 1
