# Supplementary figures and images for: Elucidating the cellular mechanism for E2-induced dermal fibrosis
Source: Arthritis Res Ther. 2021 Feb 27;23:68. doi: 10.1186/s13075-021-02441-x (PMC7913437; doi:10.1186/s13075-021-02441-x)

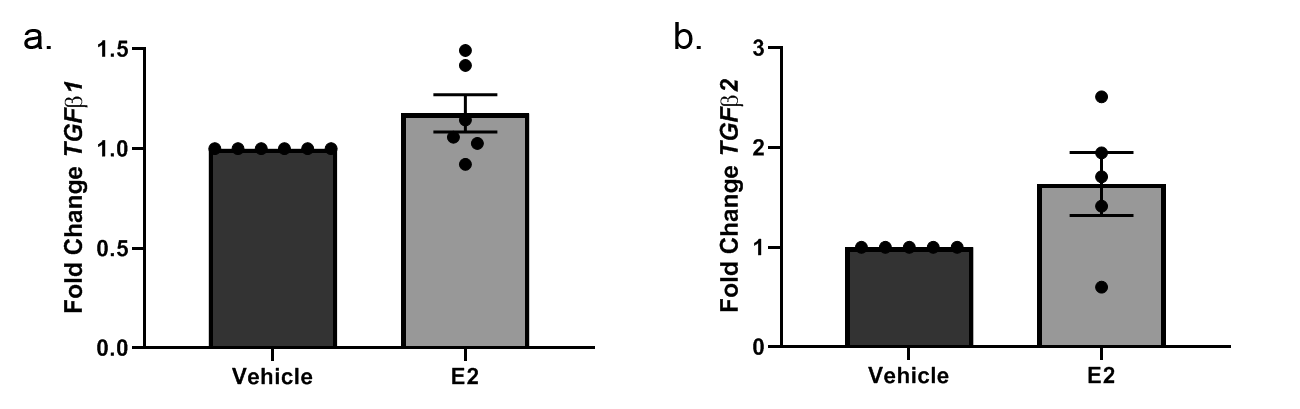

Supplement: Supplementary file 1 — Additional file 1: a-b. Steady-state mRNA levels of TGFβ1 and TGFβ2 48 h post vehicle vs. E2 stimulation in vitro. Steady state mRNA levels of TGFβ1 (a), TGFβ2 (b). Normalized to B2M. Data shown are from ≥5 independent experiments using dermal fibroblasts from 5 different donors. Bars = mean +/− SEM. Statistical test: Two-tailed, parametric, paired t-Test. [file 13075_2021_2441_MOESM1_ESM.tif]

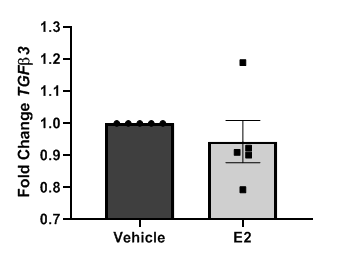

Supplement: Supplementary file 2 — Additional file 2. Steady-state mRNA levels of TGFβ3 24 h post vehicle vs. E2 stimulation in vitro. Normalized to B2M. Data shown are from ≥5 independent experiments using dermal fibroblasts from 5 different donors. Bars = mean +/− SEM. Stastical test: Two-tailed, parametric, paired t-Test. [file 13075_2021_2441_MOESM2_ESM.tif]

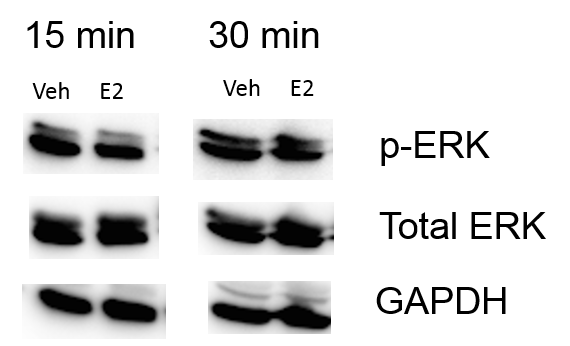

Supplement: Supplementary file 3 — Additional file 3. Immunoblot of p-ERK1/2:total ERK after 15 or 30 min of E2 stimulation. Data shown are from 2 independent experiments using dermal fibroblasts from 2 different donors. [file 13075_2021_2441_MOESM3_ESM.tif]

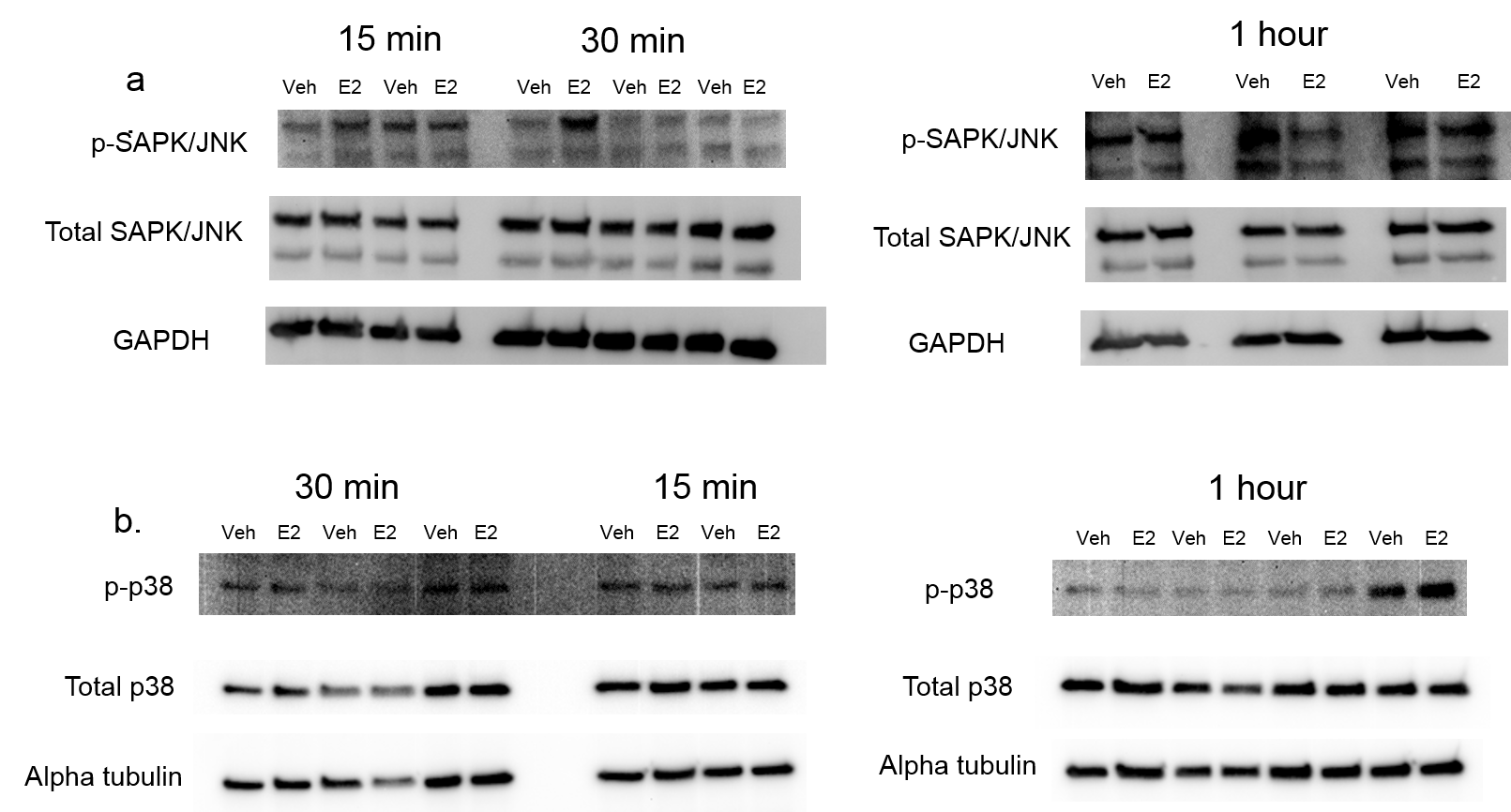

Supplement: Supplementary file 4 — Additional file 4. Immunoblots of p-SAPK/JNK:total SAPK/JNK (a) and p-p38:total p-p38 (b) 15 or 30 min or 1 h post E2 stimulation in vitro. Data shown are from ≥2 independent experiments using dermal fibroblasts from 2 different donors. [file 13075_2021_2441_MOESM4_ESM.tif]

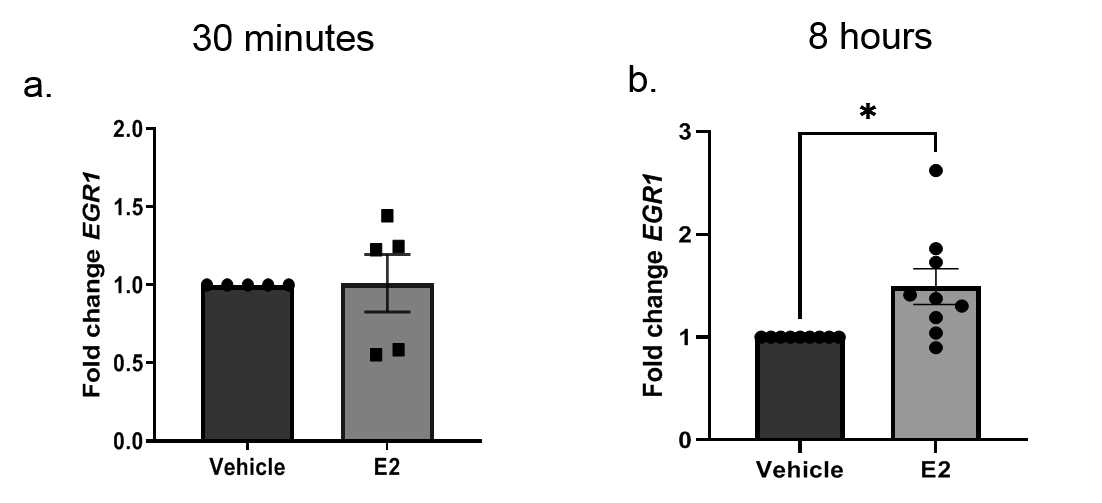

Supplement: Supplementary file 5 — Additional file 5. Steady-state transcript levels of EGR1 30 min (a) and 8 h (b) postvehicle vs. E2 stimulation in vitro. Normalized to GAPDH. Data shown are from 5 independent experiments using dermal fibroblasts from 5 different donors. 2 donors were measured in triplicate in (b). Bars = mean +/− SEM, Statistical test: Two-tailed, parametric, paired t-Test. *p ≤ 0.05 [file 13075_2021_2441_MOESM5_ESM.tif]

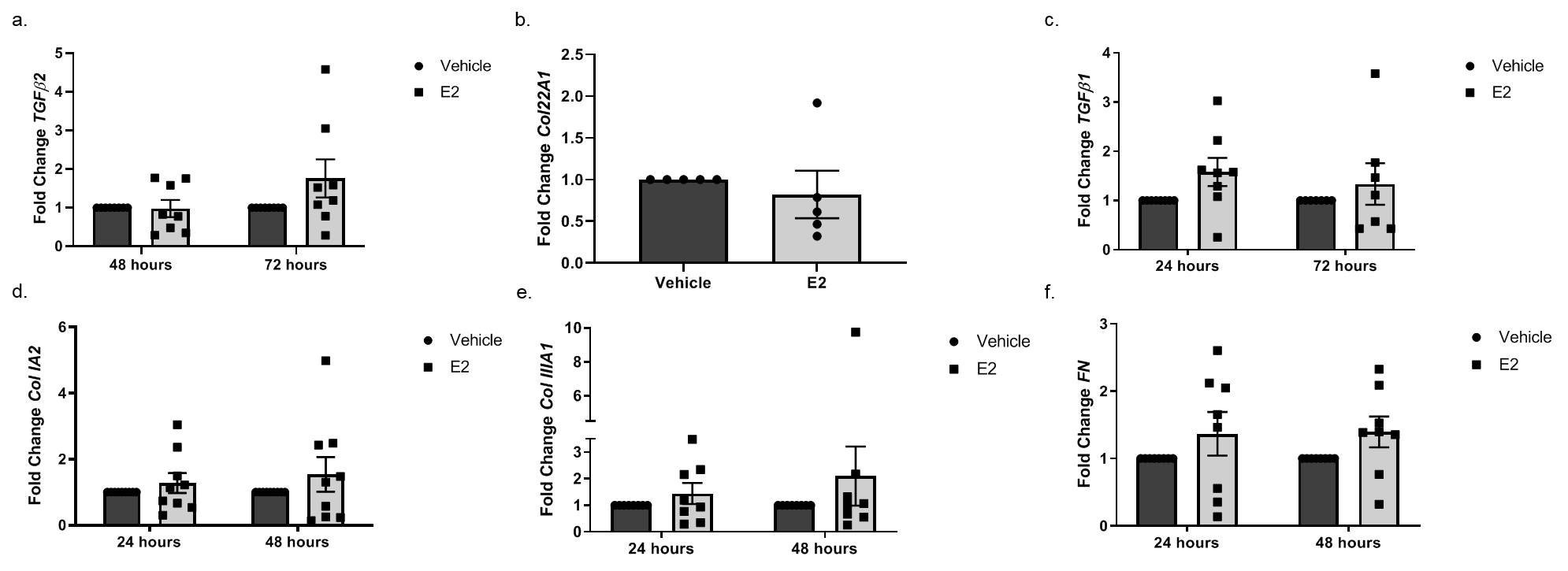

Supplement: Supplementary file 6 — Additional file 6. Steady-state mRNA levels of TGFβ2 (a), Col22A1 (b), TGFβ1 (c), Col I IA2 (d), Col IIIA1 (e), and FN (f) ex vivo. Measured at 48/72 (a), 72 (b), 24/72 (c), 24/48 (d-f) hours post vehicle vs. E2 stimulation ex vivo. Bars = mean +/− SEM. Normalized to B2M (a-e) and GAPDH (f). Data shown are from ≥8 independent experiments using dermal fibroblasts from ≥8 different donors. Statistical test: Two-tailed, parametric, paired t-Test (a-d, f) and Wilcoxon matched-pairs signed rank test (e). [file 13075_2021_2441_MOESM6_ESM.tif]

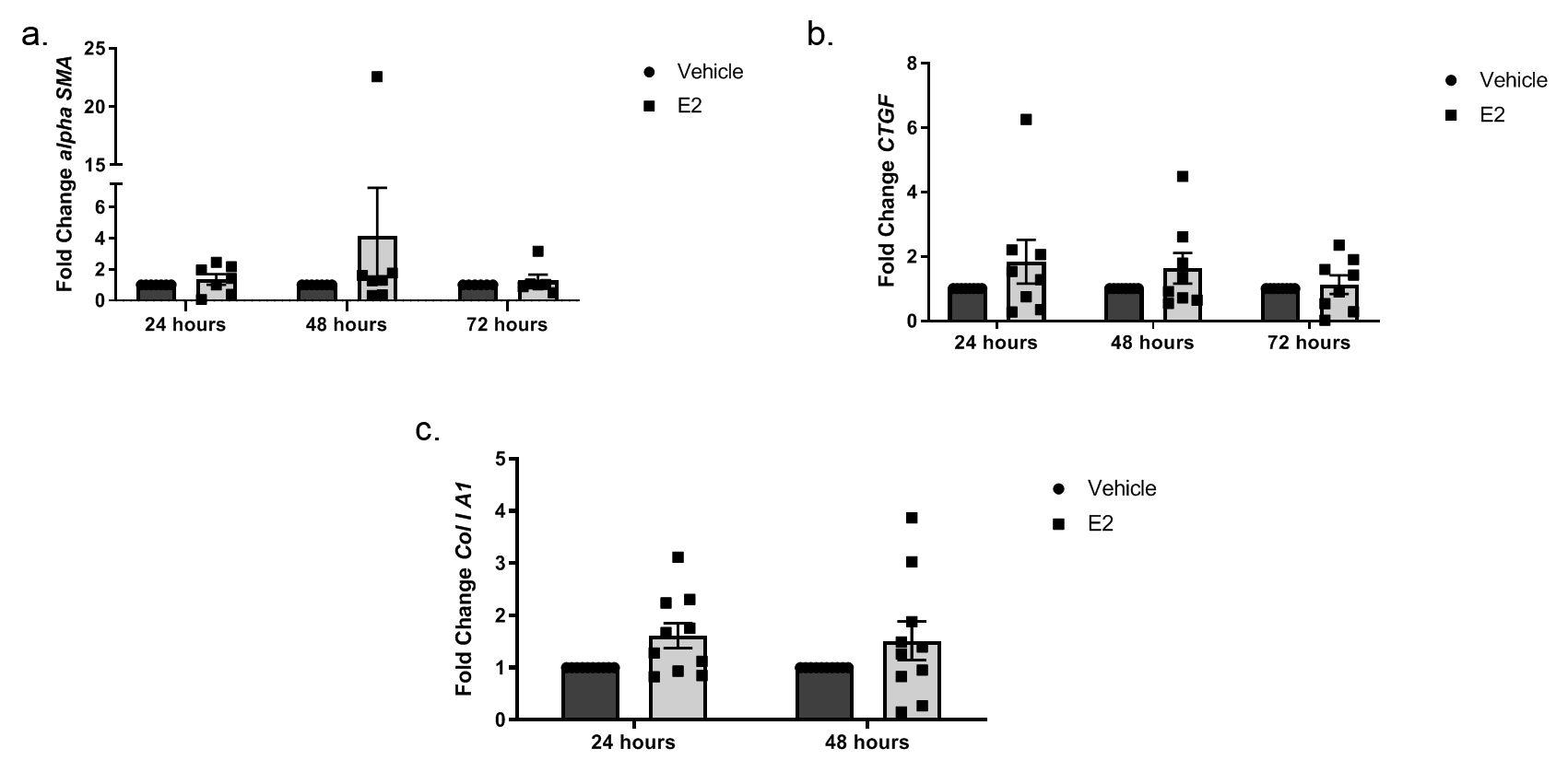

Supplement: Supplementary file 7 — Additional file 7. Steady-state mRNA levels of alpha SMA (a), CTGF (b), Col IA1 (c) ex vivo. Measured at 24–72 h (a-b) and 24–48 h (c) hours post vehicle vs. E2 stimulation ex vivo. Bars = mean +/− SEM. Normalized to B2M (a-c). Data shown are from ≥6 independent experiments using dermal fibroblasts from ≥6 different donors. Statistical test: Two-tailed, parametric, paired t-Test. [file 13075_2021_2441_MOESM7_ESM.tif]
